# A Review of Various Antioxidant Compounds and their Potential Utility as Complementary Therapy in Multiple Sclerosis

**DOI:** 10.3390/nu11071528

**Published:** 2019-07-05

**Authors:** Elzbieta Dorota Miller, Angela Dziedzic, Joanna Saluk-Bijak, Michal Bijak

**Affiliations:** 1 Department of Physical Medicine, Medical University of Lodz, Pl. Hallera 1, 90-647 Lodz, Poland; 2 Neurorehabilitation Ward, General Hospital no III, Milionowa 14, 90-001 Lodz, Poland; 3 Department of General Biochemistry, Faculty of Biology and Environmental Protection, University of Lodz, Pomorska 141/143, 90-236 Lodz, Poland

**Keywords:** multiple sclerosis, oxidative stress, dietary antioxidants, antioxidant complementary therapy

## Abstract

Multiple sclerosis (MS) is a complex disease of the central nervous system (CNS). The etiology of this multifactorial disease has not been clearly defined. Conventional medical treatment of MS has progressed, but is still based on symptomatic treatment. One of the key factors in the pathogenesis of MS is oxidative stress, enhancing inflammation and neurodegeneration. In MS, both reactive oxygen and nitrogen species are formed in the CNS mainly by activated macrophages and microglia structures, which can lead to demyelination and axon disruption. The course of MS is associated with the secretion of many inflammatory and oxidative stress mediators, including cytokines (IL-1b, IL-6, IL-17, TNF-α, INF-γ) and chemokines (MIP-1a, MCP-1, IP10). The early stage of MS (RRMS) lasts about 10 years, and is dominated by inflammatory processes, whereas the chronic stage is associated with neurodegenerative axon and neuron loss. Since oxidative damage has been known to be involved in inflammatory and autoimmune-mediated processes, antioxidant therapy could contribute to the reduction or even prevention of the progression of MS. Further research is needed in order to establish new aims for novel treatment and provide possible benefits to MS patients. The present review examines the roles of oxidative stress and non-pharmacological anti-oxidative therapies in MS.

## 1. Introduction

Multiple sclerosis (MS) is a complex neurodegenerative disease with a heterogeneous clinical course that is typified by repetitive relapses and/or progression. The onset of MS has been typically observed in individuals aged from 20 to 40 years. In MS, some pathophysiological processes in the central nervous system (CNS) can be observed, including chronic inflammation, oxidative stress, blood–brain barrier (BBB) disruption, axonal and neuronal damage, demyelination, remyelination, and repair systems [1,2,3]. The demyelination of axons is manifested by blocked impulse conduction and permanent inflammation processes. Although the pathogenesis of MS is still not completely known, it is described as an inflammatory demyelinating disease, and CNS axonal damage directly correlates with the intensification of inflammatory processes and oxidative stress [4]. In addition to demyelination processes, the progressive gradual loss of oligodendrocytes responsible for remyelination processes and axon survival is a key feature of the pathology of MS. Both chronic and recurrent microglial inflammation affect demyelination and axonal degeneration. Antigen-presenting cells (APCs) can up-regulate and down-regulate expression of the immune response, and it is considered that the microglia CNS is a typical environment for their development. It has long been known that this is an autoimmune disease; however, the molecular processes leading to axonal and neuronal damage in MS are still not understood [5]. MS is difficult to monitor due to the multiplicity of its clinical symptoms, rate of progression, and various responses to therapy, which reflect the existence of several pathogenic mechanisms. Due to its highly heterogeneous nature and unpredictable clinical course, it is characterized by relapses and remissions of clinical symptoms, with a gradual accumulation of impairment as a result of incomplete recovery during remission. The National Multiple Sclerosis Society (NMSS) Advisory Committee on Clinical Trials in MS defined in 1996 four clinical types of MS: relapsing–remitting (RR), secondary progressive (SP), primary progressive (PP), and progressive–relapsing (PR) [6]. However, in 2014, Lublin et al. proposed a modification of MS classification, dividing MS types into Relapsing MS (RRMS), and Progressive MS (SPMS and PPM), with active or inactive subtypes in both. An important modification of these phenotypes is the assessment of disease activity, as defined by the clinical assessment of relapse occurrence or lesion activity detected by CNS imaging. This modification is the most current, and makes it easier to classify cases of MS. Despite this, the terms created in 1996 are still used in the literature today [7]. The most general disease course (over 80% of MS patients) is the RR type, which is characterized by variable neurological symptoms and either complete or incomplete recovery in the remission stage. In RR, inflammatory processes associated with edema and the physiological actions of cytokines and oxidative stress dominate during relapse. More than half of the individuals who are in the RR stage of MS subsequently develop the SP form of MS, following the accumulation of neurological deficits [8]. Around 15–20% of patients have PPMS, which is characterized by the slow progression of neurological indicators from the onset of MS. Both PP and SP are characterized by gradual axonal injury. Axonal loss is the pivotal factor responsible for the conversion from RRMS to a progressive form of the disease, progressive–relapsing MS (PRMS). This is a small percentage of patients (no more than 5%), constituting a subset of PPMS after the adoption of the new classification in 2014. This rare form represents patients who experience steady progression from onset, but also have occasionally relapsed episodes of disease [6].

The aim of this review is to provide information about the various antioxidant compounds and their potential role as complementary therapy in multiple sclerosis. This review consists of 160 literature positions, including three websites. The cited works date from 1995 to 2019. The most frequently cited works are current: 2015–2019. The search for peer-reviewed articles was conducted using PubMed, Sage Journals, and SCOPUS. Google Scholar was also utilized to locate open-access articles. The following search terms were used to locate articles specific to this study: neurodegeneration, multiple sclerosis antioxidant compounds, dietary antioxidants, antioxidant complementary therapy, and oxidative stress. Variations of these terms were used to ensure exhaustive search results. After identifying all the keywords, synonyms, and phrases within a search, we used the Boolean operators ’AND’ and ’OR’ (e.g., ’oxidative stress’ AND ’multiple sclerosis’).

## 2. Pathogenesis of MS

The etiology of MS remains unknown, but it is most probably a combination of genetic, autoimmunological, and environmental factors affecting disturbed immune response. MS is an autoimmune disease with a chronic inflammation stage. In general, the pathomechanism of MS is based on the alterations of the immune system, disturbances of BBB, infiltration of large amounts of leukocytes, demyelination, and consequently leads to the death of axons/neurons. An important element of the CNS is BBB and the cerebrospinal fluid barrier (BCSFB), which regulates the active transport of molecules and cells between the circulatory and nervous systems. Both barriers are responsible for the regulation of CNS metabolic homeostasis. BBB consists mainly of matrix components and several cell types characterized by tight junctions (e.g., endothelial cells, microglia, astrocytes, and pericytes) [9]. It is believed that T lymphocytes play a major role in the initiation and propagation of MS. Autoreactive T cells infiltrate the CNS and release from their granules a large amount of pro-inflammatory cytokines that activate macrophages, leading to the inflammation of white matter, and consequently to myelin sheath destruction. A strong autoimmune reaction induces a massive influx of pro-inflammatory cells into disturbed BBB and stimulates the microglia cells to the destruction of the myelin and to forming demyelinating lesions. The chronic activation of microglia cells (present in brain) facilitate increased BBB permeability, causing the enhancement infiltration of peripheral macrophages. Furthermore, the stimulation of T helper (Th) lymphocytes (presumably Th1, mainly supporting cellular response) and their synthesis of lymphokines, such as interferon-γ (IFN-γ) and interleukin-2 (IL-2), induce B lymphocytes to transform into plasma cells. In this form, B cells can produce autoantibodies that are responsible for the neurodegenerative process, leading to the breakdown of the myelin sheath of the nerve fibers. The ratio of T:B cells under physiological conditions in peripheral blood is 9:1. During the development of autoimmune disease, such as MS, the number of B cells is significantly augmented [10]. The emerging immune response is regulated by subpopulations of T lymphocytes, including CD4+ (Th), CD8+ (cytotoxic T cells), and natural killers cells (NKs) (CD16+/CD56+). The level of regulatory T cells (Tregs) (CD4+/CD25+) is mainly responsible for intensifying the immune system response against their own antigens. Tregs inhibit humoral (production of antibodies) and cellular (cytotoxic effect) immune responses. Their actions prevent the excessive development of autoimmune reactions. There are studies that have reported that T cells display higher adhesive properties than B cells. This may mean that T cells play the largest role in the area of developing inflammation; however, B cells represent almost 40% of all CNS infiltrating lymphocytes. The BBB permeability may be caused by increased blood platelets activity, which enhances mainly T cells’ adhesion to endothelial cells and stimulates the formation of platelet–leukocytes complexes [11]. In both an MS and animal model of experimental autoimmune encephalomyelitis (EAE), neuroinflammation starts with an intensify infiltration of CD4+ T cells into CNS through the BBB. The process of T-cell activation in the CNS occurs in several stages through the mechanism of antigen presentation (immunogenic peptides) via MHC class II molecules on the surface of dendritic cells. Then, T cells recognize the complex of the antigenic determinant formed with MHC molecules due to the specific TcR receptor (T cell receptor). Activated CD4+ T cells are differentiated into Th1 and Th17 cells, which secrete a large amount of pro-inflammatory cytokines that participate in demyelination and axonal/neuronal degeneration. Accumulating evidence from studies on EAE model and MS patients indicates an important role of Th17 cells in mediating autoimmune neuroinflammation [5].

## 3. Oxidative Stress in MS

Oxidative injury is involved in cell degeneration in all stages of MS, i.e., relapse and progression, in which it can be recognized as the presence of active and slowly-expanding lesions, in both the white and grey matter [12,13,14,15,16,17]. Oxidative stress is one of the primary factors of cell injury in the aging CNS and chronic inflammation, as well as in vascular and neurodegenerative diseases, including all stages of MS. Cellular response due to tissue damage also include functional changes in the vascular endothelium, immune cell recruitment, migration, and the proliferation of microglial cells and astrocytes, and the production of pro-inflammatory cytokines, as well as phagocytosis. During the repair processes of damaged nervous tissue, a cascade of side effects occurs that affects the excessive production by phagocytes, inflammatory cytokines, and reactive oxygen species (ROS), as well as reactive nitrogen species (RNS). The excessive production of ROS and RNS leads to leukocyte migration, oligodendrocyte damage, and axonal injury. Oxidative stress causes damage to cardinal cellular components proteins, lipids, and nucleic acids, resulting in cell death by apoptosis. The accumulation of ROS and RNS may cause mutation in RNA, nuclear DNA, and mitochondrial DNA (mtDNA), inducing the up-regulation and down-regulation of key genes of cellular metabolism, and not only. Free radical production can activate major factors, such as nuclear factor kappa B (NFκB), that up-regulate pro-inflammatory gene expression. Therefore, oxidative stress is involved in the functioning of different types of CNS cells, and affects some cellular components that exacerbate inflammation and neurodegenerative processes [1,2,8,18,19,20,21]. ROS and RNS are created at a high level in the CNS of MS patients, mainly in specific brain cells, such as astrocytes, activated macrophages, and microglia. The most numerous cells in the nerve system are astrocytes, which constitute about 90% of the CNS population. They participate in the most important physiological functions, which include homeostasis and maintaining BBB integrity, myelination, and functions regulating the neurons’ neurotransmission [22,23]. The inflammatory cytokines such as tumor necrosis α (TNF-α) and IFN-γ are responsible for the production of nitric oxide (NO) in the CNS, mainly by macrophages and microglial cells following the induction of NO synthase (NOS). NO production in the brain is regulated by NOS, which is present in various cell types. Neurons in physiological conditions generate NO at a concentration of about 30 nM, while microglial cells and astrocytes can increase NO production to 100-fold higher concentration. NO can react rapidly with superoxide anions (O_2_^-•^) to create RNS, such as peroxynitrite (ONOO^-^), which is a very strong oxidant that has been associated with neuronal loss, and is considered as a pathogenic agent in MS [24,25]. Studies have shown that NOS is up-regulated in inflammatory lesions, and that NO and its derivative ONOO^-^ inhibits mitochondrial respiration [26].

During relapse in the early stage of MS, oxidative stress is the key factor involved in inflammation and oxidative microglia activation. However, during the progressive stage, aging processes such as chronic mitochondrial injury or the accumulation of metals, such as iron (catalytic for the free radical reactions) in the brain are also enhanced, and can trigger the formation of demyelinating lesions [27]. Neurons are highly dependent on oxidative energy metabolism and very sensitive to bioenergetic fluctuations. In order to fuel sodium–potassium ATPase or a sodium potassium pump, axons consume a huge amount of ATP. This action aims to remove sodium ions that pass to the axon during impulse activity. Therefore, the greatest number of mitochondria is located at presynaptic sites, where the energy request is especially high. Particularly, the most exposed to changes in the shape and function of the mitochondria are the brain and CNS [28]. Oxidative stress contributes to changes in mitochondrial functioning, mainly by interfering in various components of the respiratory chain [29], the most vulnerable of which to ROS activation is the heme-containing molecule cyclooxygenase 1 (COX-1), which is also known as complex IV [30]. These mechanisms affect energy metabolism and decrease adenosine triphosphate (ATP) levels, and gradually enhance neuronal dysfunction or neurodegeneration. There are also other enzymatic pathways, such as xanthine oxidase, NADPH (nicotinamide adenine dinucleotide) oxidase, lipoxygenase (LO), as well non-enzymatic mechanisms by the autoxidation of catecholamines that cause the production of free radicals [31].

The increased energy demand of demyelinated axons suffering mitochondrial dysfunction is particularly harmful in demyelinating diseases, such as MS. In patients with MS, mitochondrial structural changes, different mitochondrial gene expression and enzyme activities, increased ROS production, and oxidative damage have been observed [32,33,34]. As discussed above, both oxidative and mitochondrial injury primarily influence the cell processes of neurons and glia, causing disturbances in cellular communication before real cell loss is observed. It can be concluded that the original trigger of oxidative injury induces various cascades of molecular processes inducing degeneration and cell death, which vary depending on cell type [35].

## 4. Antioxidant Compounds as Complementary Therapy in MS

**Figure 1:**
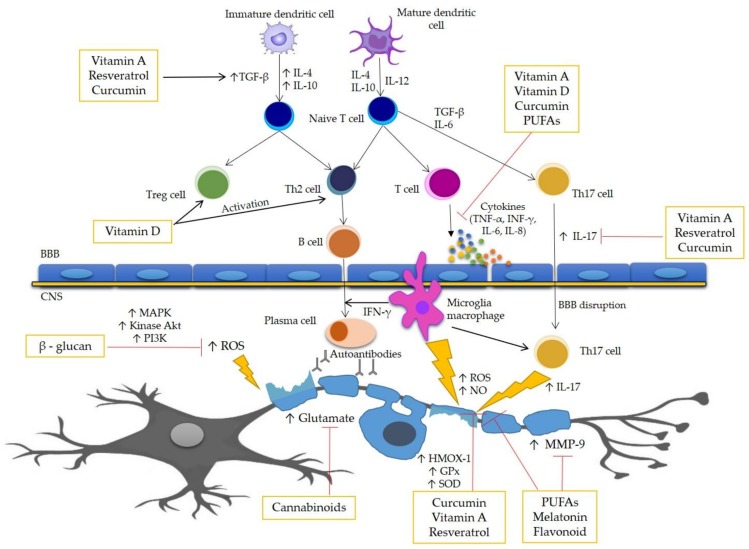
Pathomechanism of MS and potential action site of bioactive compounds. One of the major factors in the pathogenesis of MS is oxidative stress, enhancing inflammation and in consequence causing damage of the myelin sheath and death of the neurons. The course of MS is associated with the secretion of many inflammatory and oxidative stress mediators, including cytokines, such as IL-1β, IL-6, IL-17, TNF-α, and INF-γ. The process of demyelination is caused by the action of many cells of the immune system mainly by macrophages, B-cells, and T cells, as well as the increased permeability of the blood–brain barrier (BBB). This figure represents the scheme of the potential action site of the bioactive antioxidant compounds.

Several diseases have been associated with oxidative stress, suggesting that it is one of the factors that could be a trigger for many diseases, and that antioxidant therapy could be an effective additive therapeutic treatment. However, while basic research and preclinical studies support this point of view, clinical studies still produce controversial results. The pathophysiological complexity of ROS/RNS signaling in humans is most likely due to the presence of comorbidities. Several studies have shown that antioxidant therapy is beneficial in vitro and in vivo in animal models for MS. Therefore, it is considered that antioxidant therapy may represent an attractive treatment of MS. This work presents several selected compounds that are key elements of antioxidant therapy for people with MS. The anti-oxidative properties of described dietary compounds and standardized extracts used in treatment of MS are represent in Table 1. In this work, there is a figure (Figure 1) at the end of Section 4, which contains a demonstration of the pathomechanism of MS and the potential bioactive action of the above-described compounds.

### 4.1. Curcumin

Curcumin (diferuloylmethane) is the main active component of turmeric, which was derived from the rhizome of the East Indian plant, *Curcuma longa*. Commercially available curcumin is a crystalline compound with a bright orange-yellow color, containing three major components: curcumin (77%), demethoxycurcumin (17%), and bisdemethoxycurcumin (3%), which are known as curcuminoids [36]. Curcumin has lipophilic features and is able to pass through all cell membranes, and hence is able to exert intracellular effects [37]. It is able to cross the BBB and regulate the homeostasis of the CNS microenvironment by inhibiting the key pro-inflammatory cytokine secretion pathway [38]. Curcumin has broad therapeutic potential, possessing antioxidant and anti-inflammatory properties and allowing the direct detoxification of ROS. Its anti-inflammatory effects and neuroprotective efficacy have been assessed in many neurodegenerative diseases, including Alzheimer’s disease (AD), Parkinson’s disease (PD), and MS [39]. Curcumin has been shown to suppress the activation of the NFκB pathway and inhibit the production of inflammatory cytokines by activated monocytes and macrophages, including COX-2, inducible nitric oxide synthase (iNOS), monocyte chemoattractant protein 1 (MCP-1), macrophage inflammatory protein (MIP-1α), and interleukins: IL-1, IL-2, IL-6, IL-8, and IL-12. Curcumin blocks the cytokines mediating NFκB pathway activation by inhibiting protein kinase B (AKT kinase) and IκBα (inhibitor of kappa B) via various inflammatory stimuli; it also down-regulates the expression of NFκB regulated gene products, including IL-1β, IL-6, IL-8, IL-17, TNF-α, MIP-1α, prostaglandin E2 (PGE2), C-reactive protein (CRP), and C-X-C chemokine receptor type 4 (CXCR-4), as well as others induced by inflammatory stimuli. Being an NFкB pathway inhibitor, curcumin effectively prevents disruption of the BBB induced by Th17 cells [40]. Other research conducted on EAE mice indicates that immunization with myelin basic protein 68–86 (MBP 68–86) peptide emulsified in complete Freund’s adjuvant (CFA) with curcumin (100 and 200 mg/kg of body mass) significantly ameliorated the clinical severity of EAE in animals, and was associated with a significant decrease of pro-inflammatory molecules, including transforming growth factor β (TGF-β), IL-6, IL-17, IL-21, and signal transducer and the activator of transcription 3 (STAT-3) compared with healthy controls [41]. Curcumin activates many antioxidant systems, such as nuclear factor erythroid 2-related factor 2 (Nrf2), hemeoxygenase1 (HMOX-1), heat shock proteins 70 (Hsp70s), and thioredoxin, causing neuroprotective effects and representing an innovative approach to therapeutic intervention in neurodegenerative diseases [42]. It is well known that the accumulation of transition metals in the brain, such as Mn^2+^, Fe^2+^, Cu^2+^, and Zn^2+^, intensify oxidative stress and are involved in some neurodegenerative disorders. In MS, curcumin can act as a versatile agent to scavenging ROS, blocking protein aggregation, and chelating metal ions [43,44]. There are two tautomeric forms of curcumin: β-diketone (CurK) and β-keto-Enol (CurE). CurE has greater antioxidant activity than CurK. CurK are likely to form metal chelates and scavenge the active free radicals, which may be produced by the metal ions. The binding of transition metals by curcumin and the formation of stable inactive complexes, especially with ferrous ions (Fe^3+^), may be one of the key ways to prevent the brain from neurodegenerative disorders [45]. Experiment on SJL/J mice showed that curcumin inhibits IL-12-induced T-cell responses and the tyrosine phosphorylation of transcription factors: STAT-3, STAT-4, Janus kinase 2 (JAK2), and tyrosine-protein kinase 2 (TYK2) in T cells. They demonstrated that curcumin is a potent regulator of IL-12 production/signaling and the differentiation of Th1. Moreover, they also revealed that it decreased the clinical severity and inhibition the development of paralysis [46]. Researchers Mohajeri et al. showed an efficient therapeutic effect of polymerized form of nanocurcumin (PNC) on adult female Lewis rats (an EAE animal model), and showed that PNC is a potential anti-inflammatory and anti-oxidative stress agent, with significant effects on the EAE scores and myelin repair mechanisms. They showed that PNC diminishes pro-inflammatory genes expression: MCP-1, IL-1, IL-17, NFκB, and augments the expression of anti-inflammatory gene markers: IL-4, forkhead box P3 (Foxp3) (an important transcription factor that belongs to the forkhead/winged-helix family of transcriptional regulators) and TGF-β. They also indicated that PNC modulates the expression of oxidative stress marker genes, including increased levels of iNOS, HMOX-1, and Nrf2 mRNA in the lumbar spinal cord in Lewis rats [47].

### 4.2. Melatonin

Melatonin (*N*-Acetyl-5-methoxytryptamine) is produced naturally by the pineal gland during the night in response to darkness. The daily production of melatonin in a young adult is about 30 μg (mean concentration 10 pg/mL per day, including the maximum nocturnal concentration around 200 pg/mL) [48]. Unfortunately, around the age of 40, the level of melatonin secretion clearly decreases, which is mainly due to the progressive mineralization of the pineal gland structures. Melatonin is currently being supplemented, but the bioavailability of the compound taken via the gastrointestinal tract is around 15% [49]. It is very important to have a proper diet that ensures the synthesis of melatonin at the appropriate level. Melatonin is formed from exogenous tryptophan, and its transformations include four stages, in the course of which serotonin is formed. Therefore, tryptophan is the precursor of both serotonin (5-hydroxytryptamine, 5-HT) and melatonin and a lack of tryptophan in the diet may lead to disturbances in the sleep rhythm (lack of melatonin), as well as mood disorders (lack of 5-HT) [50]. Tryptophan is commonly found in foods that contain protein. Foods that naturally boost 5-HT/melatonin concentrations, mainly include meat (mainly poultry), oily fish such as salmon, eggs, milk, seeds, nuts, almonds, and soy products. However, according to research, protein-rich products hinder the proper absorption of tryptophan. It should be noted that only carbohydrate-containing foods that cause insulin to be released into the blood allow the body to properly assimilate this exogenous amino acid. One of the better choices is bananas, which are abundant in tryptophan, and because they largely contain sucrose, glucose, fructose, and starch, it can be effectively assimilated [51]. An investigation of the effect of dietary tryptophan enrichment (30–40 mg of tryptophan/kg body weight) of whey protein on affective and cognitive functions in MS patients showed that the tryptophan-fortified diet increased memory processes without ameliorating the mood states. An intake of tryptophan-enriched whey protein acutely enhances the recall of positive loaded words in MS patients [52]. Melatonin plays an important role in the regulation of the circadian rhythm, sleep, and mood; however, it also has immunomodulation properties. Melatonin is produced by various cells such as bone marrow cells, astrocytes, macrophages, T cells, and fibroblasts [53]. Melatonin produced by the pineal gland could be an effective alternative for treating cerebral edema, and as a highly lipophilic molecule, melatonin can freely cross the BBB, thus reducing the volume of edematous sites. Unfortunately, the molecular mechanism of action remains unclear [54,55]. It is also suggested that melatonin can protect against IL-1β-induced BBB dysfunction and hyperpermeability in vitro; firstly via matrix metallopeptidase 9 (MMP-9) inhibition and secondly by maintaining tight junctional and cytoskeletal integrity. Moreover, the levels of active MMP-9 in the serum and cerebrospinal fluid (CSF) of MS patients may serve as indicators for the monitoring of disease activity [56]. Experiments conducted on rat brains demonstrated that melatonin up-regulates enzymatic anti-oxidative defensive systems, including stimulating the synthesis of superoxide dismutase (SOD) and glutathione peroxidase (GPx) [57]. Miller et al. have demonstrated that oral melatonin supplementation (10 mg daily for 30 days) increased the concentration of SOD and GPx, as well as decreased the concentration of a typical marker of oxidative stress—malondialdehyde (MDA)—in erythrocytes of patients with SPMS (*n* = 16) [54]. The results obtained by Melamud et al. on MS patients (*n* = 13) during IFN-β treatment demonstrated significantly decreased levels of 6-sulphatoxymelatonine (6-SMT) and disrupted circadian regulation. Their findings suggest that the dysregulation of melatonin secretion in MS may be influenced by IFN-β treatment. That result needs to be more specifically analyzed in terms of the role of neurohormones and their cross-regulation with immune mediators. The main limitation of this study is the small size of the group; this result requires further analysis of a larger study group [58].

### 4.3. Vitamin D

Vitamin D is a steroidal compound that is metabolized in the skin, liver, and kidneys. To achieve biological activity, vitamin D needs to be metabolized within the body to the hormonally-active form, which is known as 1,25-dihydroxycholecalciferol (1,25-(OH)_2_D3). This is a two-step transformation process. First, vitamin D3 is hydroxylated by 25-hydroxylase–CYP2R1 to 25-hydroxycholecalciferol (25(OH)D3), which is a substrate for 1-alpha-hydroxylase, yielding 1,25-(OH)_2_D3. The first step occurs mainly in the liver, while the second step occurs principally in the kidneys. However, other tissues including various epithelial cells, immune cells, and the parathyroid gland also possess the ability to perform this hydroxylation [59]. The major circulating form of vitamin D3 is 25(OH)D3; however, vitamin D exists in two forms: vitamin D2 (ergocalciferol) and vitamin D3 (cholecalciferol). Vitamin D3 is mainly found in fatty animal-sourced foods, such as fish oil and egg yolk, while vitamin D2 comes from plant-sourced foods, such as mushrooms (grown in UV light), fortified foods, and dietary supplements [60]. Currently, there is very little information about vitamin D2 in neurodegeneration, including MS. The study carried out on adult patients with clinically active RRMS (*n* = 23), who were randomized to six months of a double-blind placebo-controlled high dose of vitamin D2 (6000 IU/day), showed that high-dose vitamin D2 compared to low-dose supplementation (1000 IU/day), was similarly not effective in reducing MRI lesions in RRMS patients [61]. Besides maintaining bone health and calcium metabolism, vitamin D3 is involved in a number of functions through its action on vitamin D receptors (VDR), which are present in most cells (microglia, activated monocytes, B and T cells) and tissues (skin, intestine). Vitamin D3 may play an immunomodulatory role in the CNS though VDR activation, which is known to alter the transcription, proliferation, and differentiation of immune cells [62]. Epidemiological studies conducted on MS patients (*n* = 256) have revealed a relationship between geographical location and the occurrence of MS. One potential explanation for this phenomenon is that MS susceptibility is associated with exposure to sunlight and the subsequent production of vitamin D. Following the hypothesis, vitamin D supplementation and higher circulating vitamin D levels are associated with a decreased risk of MS [63]. Moreover, ultraviolet radiation (UVR) exposure causes systemic immune suppression in humans, which is related to the delay, or even halting of progression in clinical and imaging manifestations of MS. As demonstrated in the studies, it is possible that additional ultraviolet B (UVB) phototherapy patients might have possible benefits in MS patients. The mechanisms by which UVB exposure may have regulated MS development are unknown, but it seems likely that it is associated with immunoregulation (even by increasing Tregs) by both vitamin D-dependent and vitamin D-independent pathways [64]. Vitamin D has been also shown to have immunomodulatory effects, decreasing Th1 activity and increasing Th2 and Tregs activity, and may therefore play a role in the etiology of MS. A study conducted by Salzer et al. on MS patients (*n* = 192) reported that the presence of 25(OH)_2_D3 levels greater than 75 nmol/L in prospectively-collected samples were associated with a 61% decrease in the risk of occurrence of MS [65]. It is recognized that vitamin D deficiency can be considered an independent factor that is associated with the disability of the MS patients and progression of the disease. Oliveira et al. showed that MS patients (*n* = 137) with vitamin D deficiency (<50 nmol/L) had heightened disease progression as evaluated by the Multiple Sclerosis Severity Score (MSSS) and Expanded Disability Status Scale (EDSS) compared to those MS patients with sufficient vitamin D3 status (>50 nmol/L). They also demonstrated that MS patients with vitamin D deficiency have lower nitric oxide metabolites (NOx) compared to MS patients with >50 nmol/L vitamin D status (17.05 μM ± 12.35 versus 34.84 μM ± 27.39, respectively). It has been suggested that NO production via inducible nitric oxide synthase (iNOS) plays an important role in the pathogenesis of several systemic autoimmune disorders, include MS. The active vitamin D regulates the production of one of the oxidative stress markers—nitrogen oxide—and/or the expression of iNOS in different cells such as microglial cells, macrophages, and astrocytes [66]. Detailed studies performed on the mouse model induced EAE, and showed that vitamin D3-supplemented animals undergo intensive synthesis of 1,25-(OH)_2_D3 in the CNS, which was correlated with the suppression of EAE. This effect was observed only in females, and a decrease in CYP24A1 transcripts encoding the 1,25-dihydroxyvitamin D3-inactivating enzyme in the spinal cord was detected. However, ovariectomy abolished this protective effect. Therefore, there is possible synergy between the ovarian tissue and vitamin D3 with respect to EAE inhibition, which potentially allows the ovarian tissue to control vitamin D3 metabolism and anti-inflammatory functions in the CNS. It is probable that the female hormones, by inhibiting CYP24A1 gene expression in the spinal cord, allowed the accumulation of 1,25-(OH)_2_D3 in the CNS, which suppressed EAE by immunomodulation. It is possible that a similar gender difference in vitamin D metabolism in the CNS may occur in humans, as indicated by unexplained higher MS incidence in women than in men [67]. Meanwhile, EAE was completely prevented by the administration of exogenous 1,25-(OH)_2_D3. The protective mechanism against EAE involved a limitation of occurrence of activated autoreactive T cells in the CNS [68], the reduced accumulation of macrophage in the CNS [69], stimulating inflammatory cell apoptosis, and enhancing CNS cell survival. However, the activated inflammatory cells can produce 1,25-(OH)_2_D3, and this hormone subsequently exhibits anti-inflammatory activities directed against these cells [70]. Molecular experiments provide in vivo evidence that 1,25-(OH)_2_D3 acts directly on pathogenic CD4+ T cells and inhibits EAE via VDR in T lymphocytes. Moreover, this result is most compatible with the sensitization of encephalitogenic T-cells to CNS-derived apoptotic signals [71]. Vitamin D3 add-on treatment to IFN-β reduces magnetic resonance imaging (MRI) disease activity in MS. A one-year, double-blind, placebo-controlled, randomized study in Finland conducted on MS patients (*n* = 66) measured the safety and efficacy of vitamin D3 as an add-on therapy to IFN-β. Obtained results indicate that serum levels of 1,25-(OH)_2_D3 increased from a mean of 54 nmol/L to 110 nmol/L in the vitamin D3 group. Patients supplemented vitamin D3 also showed fewer new lesions and a significantly lower number of enhancing lesions, as well as a tendency to reduced disability accumulation [72]. In contrast, a double-blind placebo-controlled randomized clinical trial conducted on RRMS patients (*n* = 50) were randomly allocated to receive 12 months of treatment with either escalating calcitriol (a metabolically active form of vitamin D3—1,25-(OH)_2_D3) doses up to 0.5 μg per day or a placebo combined with disease-modifying therapy. In RRMS patients, the mean relapse rate decreased significantly compared to patients treated with placebo, in which the mean EDSS increased at the end of the study period. Despite this, a low dose of vitamin D3 to routine disease-modifying therapy had no significant effect on the EDSS score or relapse rate [73]. A study carried out on 324 participants with RRMS showed that patients taking IFN-β/GA simultaneously and IFN-β had a higher level of 25(OH)D. In the fingolimod (FTY) group, which was analyzed separately, they observed an approximately 50% reduction in new inflammatory events and relapses with higher 25(OH)D levels [74]. Research conducted on a cohort of 178 persons with clinically definite MS from southern Tasmania in 2002–2005 measured the serum 25(OH)D level biannually. They found that IFN-β therapy is associated with a greater production of vitamin D from sun exposure, suggesting part of the therapeutic effects of IFN-β on relapse in MS, which may be through the modulation of vitamin D metabolism [75].

### 4.4. Omega-3 Polyunsaturated Fatty Acids (Omega-3 PUFAs)

The omega-3 polyunsaturated fatty acids (PUFAs) possess potent immunomodulatory activities, with the most biologically potent omega-3 PUFAs being eicosapentaenoic acid (EPA) and docosahexaenoic acid (DHA). Animal experiments and clinical intervention studies indicate that omega-3 PUFAs have anti-inflammatory properties and may prove useful in the management of MS and other inflammatory and autoimmune diseases [76]. EPA and DHA are two forms of long-chain omega-3 PUFAs that act as immune cell modulators and have been reported to decrease the levels of pro-inflammatory cytokines secreted from stimulated peripheral blood mononuclear cells (PBMC) obtained from patients with MS. One of the key cytokines produced by macrophages and monocytes are TNF-α, IL-1β, and IL-6. A conducted study on MS patients (*n* = 20) showed that omega-3 PUFA supplementation may suppress the capacity of monocytes to synthesize IL-1 and TNF-α, and result in a decrease in the production of the inflammatory cytokines IL-1β, IL-2, TNF-α, and IFN-γ in people with MS [77]. An experiment performed on peripheral blood mononuclear cells (PBMCs) suggested that omega-3 PUFAs may have a therapeutic role in MS by modulating the immune cell production of MMP-9. In MS, MMP-9 is thought to play a pivotal role in the transmigration of inflammatory cells into the CNS by aiding in the disruption of the BBB. EPA and DHA significantly decrease the levels and activity of the MMP-9 protein, and significantly inhibit T-cell migration into the CNS [78]. Generally, PUFAs are among the molecules that are most sensitive to ROS. PUFAs, including EPA and DHA, are cellular compounds that are readily oxidized when exposed to air or dissolved in organic solvents, because they have numerous bisallylic hydrogen atoms. The in vivo anti-oxidative function of EPA probably arises through a combination of EPA and its counterpart fatty acid of the phospholipid molecules to shield membranes in advance of the effects of exogenous ROS. Research conducted by Sakai et al. on human aortic endothelial cells (HAECs) showed that EPA and DHA attenuated DNA damage independently of the DNA damage response. Moreover, EPA and DHA significantly reduced intracellular ROS under both basal condition and H_2_O_2_ stimulation. In addition, the gene expression on mRNA levels of antioxidant molecules, such as HMOX-1, thioredoxin reductase 1 (TXNRD-1), and manganese superoxide dismutase (MnSOD) were significantly increased with EPA and DHA [79]. Due to the high protective potential, the number of clinical trials on omega-3 fatty acids that are aimed at demonstrating the various beneficial activities of these compounds in MS is constantly increasing. Pantzaris et al. conducted a small-size, randomized, controlled phase II clinical trial that provided evidence for a novel nutraceutical formula based on dietary, metabolic, immunological, and neurobiological pathways possibly involved with the etiology of MS and disease progression. They showed that combining many specific active ingredients including a mixture of EPA, vitamin A, γ-tocopherol, and other specific active molecules into one stable formulation significantly reduced the annualized relapse rates and the risk of sustained disability progression without any reported serious adverse events. However, the observed magnitude of the benefit effect is not the sum of the postulated efficacy estimates of the individual ingredients [80]. In addition, other randomized trials showed that omega-3 fatty acid supplementation as an augmentation therapy for treatment-resistant depression in MS is not significantly different than placebo [81]. The expected effects were also not proved by other, randomized clinical trials conducted to determine whether omega-3 fatty acids reduce magnetic resonance imaging (MRI) and clinical disease activity in patients with multiple sclerosis, both as monotherapy and in combination with IFN-β treatment. No beneficial effect of omega-3 fatty acids on disease activity was noticed in comparison to placebo. In addition, this supplementation did not interfere with IFN-β treatment [82]. Significant limitations in clinical studies (regarding the number of enrolled patients, varied course of disease, and even using the corn oil capsules as a standard placebo which contain linoleic and oleic acid with potential anti-inflammatory properties) do not allow rejecting the importance of omega-3 fatty acids in MS, but certainly indicate the need for further reliable research.

### 4.5. Vitamin A

Vitamin A is a fat-soluble nutrient with a variety of essential functions in eyesight, skin, and immunity. The physiological actions of vitamin A are mediated by its most active metabolite, retinoic acid (RA). RA enables the modulation of plasticity and neuron regeneration in the CNS [83]. It has been found that a negative correlation exists between serum vitamin A level and the development of MS, and that MS patients possess a lower level of vitamin A in plasma [84]. In murine model of EAE (C57BL/6 mice), RA enhances the induction of Foxp3 Tregs, which are critical in maintaining immune tolerance and the homeostasis of the immune system, and inhibiting the differentiation of CD4+ T cells into Th1 and Th17 subsets [85]. The results of a clinical trial, over a six-month period, on MS patients (*n* = 39) that received vitamin A also confirmed the up-regulation of TGF-β and Foxp3 gene expression in peripheral blood mononuclear cells. By this, vitamin A restores the impaired immunity balance that is responsible for disease immunopathogenesis [86]. RA is also able to modulate the balance between Th1/Th2, which are responsible for cellular and humoral response, and Th17/Tregs, which are responsible for inflammatory equilibrium (Th17 cells produce stimulating inflammation interleukins, such as IL-17, IL-22, and IL-23; on the other hand, Tregs produce anti-inflammatory cytokines, such as IL-10 and TGF-β, suppressing immune response) [87] as well as dendritic cells (DCs) and B cell functions, and has been found to enhance tolerance and reduce inflammatory processes [88]. Vitamin A may promote the differentiation of immature CD4+ T cells away from pathogenic Th1 and Th17 phenotypes toward an immunoprotective Th2 and Tregs pattern through the modulation of the particular transcription factors that are related to these subsets [89]. In vivo and experimental studies indicate that vitamin A deficiency may play a role in the pathogenesis of MS. There is clear evidence that vitamin A can suppress EAE, inhibit the generation of cytokines (IL-1β, IL-12, TNF-α) and NO in EAE mice, and decrease the proliferation of myelin basic protein (MBP)-reactive cells [90]. The mechanism of the anti-oxidative effect of RA remains unclear, but there are studies that have shown that RA and 9-*cis* RA stimulate binding peroxisome proliferator-activated receptors (PPAR) to peroxisome proliferators response elements (PPRE), which enhances SOD-1 gene expression in rat brains. Activated PPARs are also capable of transcriptional repression through DNA-independent interactions with other transcription factors, such as NFκB signal activators and transducers of transcription STAT-1 and activating protein 1 (AP-1) signaling [91,92]. Research carried out by Ahlemeyer et al. on primary cultures from neonatal rat hippocampus clearly indicate a decline in the protein levels of SOD-1 and SOD-2 in the presence of 10 nM of RA, as well as a reduction in staurosporine-induced oxidative stress and apoptotic damage [91]. A study carried out on RRMS patients (*n* = 88) did not show any correlation between retinol and IFN-β. Moreover, an increase of 1 μmol/L of serum retinol reduced the risk of developing new enhancing lesions by 49%, new lesions by 42%, and active lesions by 46%. They indicate that vitamin A metabolites may play a role in determining the risk of developing clinical and MRI activity in MS patients [92].

### 4.6. Flavonoids

Flavonoids, also known as bioflavonoids, play an important role in the management of MS. They are colorful polyphenols with antioxidant properties found in plants, and are categorized into flavonols, flavones, flavanones, isoflavones, catechins, anthocyanidins, and chalcones based on their chemical structure. Pure flavonoids such as quercetin, lutein, genistein, and hesperetin, or their enriched extracts, can reduce the expression of pro-inflammatory cytokines, including IL-1β, IL-6, and TNF-α [93]. The flavonoid-rich extract (FRE) obtained from *Rosa laevigata* Michx fruit (RLMF) shows a neuroprotective effect against cerebral ischemia–reperfusion (I/R) that induced injury in rat brains. In addition to scavenging free radicals and reducing neuron apoptosis, the extract also inhibited neuroinflammation. In fact, treatment with FRE at doses of 50 to 200 mg/kg decreases the expression of the pro-inflammatory markers (such as NFκB, iNOS, COX-2, MMP-9, TNF-α, IL-1β, IL-4, and IL-6) and reduced the levels of the c-jun N-terminal kinase (p-JNK), protein kinase RNA-like endoplasmic reticulum kinase (p-ERK), and phospho-p38 members of the mitogen-activated protein kinase (MAPK) pathway [94,95]. The versatile mode of action of flavonoids includes scavenging the free radical, chelating metals, and silencing the pro-oxidative enzymes associated with free radical generation, such as protein kinase C, xanthine oxidase, COX, lipoxygenase, and NADPH oxidase [96]. One of the best-studied flavonoids in MS and EAE is epigallocatechin-3-gallate (EGCG). Green tea and its active ingredient, EGCG, have been shown to modulate immune cell functions and improve the condition of some autoimmune diseases, such as MS. A study conducted on EGCG inhibited immature CD4+ T cell differentiation into Th1 and Th17 effector subsets by influencing their respective signaling transducers and transcription factors. These changes have been found to exert an influence on the immune and inflammation profiles of lymphoid tissues and the CNS, such as reducing autoreactive T-cell proliferation and pro-inflammatory cytokine production, while also reducing the Th1 and Th17 subpopulations, but increasing regulatory T cell populations. These results suggest that green tea or its active components may have preventive and therapeutic potential in dealing with T-cell-mediated autoimmune diseases [97]. In 2004, Aktas et al. reported that oral-applied EGCG suppressed inflammation in vivo through the inhibition of synthesis TNF-α in T cells, and effectively protecting against the CNS relapsing during autoimmune disease in EAE mice. EGCG reduced axonal damage and neuronal cell death by directly targeting ROS formation. The decreased proliferation of CD4+ T cells incubated with EGCG could be linked to its interference with the cell cycle, NFκB activation, and protein degradation pathway (proteasome) [98]. The supplementation of healthy volunteers with 500 mg per day of catechins for four weeks decreased plasma oxidized low-density lipoprotein (LDL) by 18% compared to control [99]. EGCG is a powerful free radical scavenger that sheltered neurons from the oxidative damage. The antioxidant activity of EGCG is due to the presence of phenolic groups that are sensitive to oxidation and can generate quinone. The anti-oxidative effect is further augmented by the presence of the trihydroxyl structure. Further, EGCG may ameliorate lipid infusion-mediated insulin tolerance, which is related to the enhanced expression of antioxidant enzymes, such as SOD and GPx by EGCG in vivo [100]. When treating human mammary epithelial cells with EGCG, the increased expression of HMOX-1 and SOD was recorded. This effect was reduced by the siRNA-mediated disruption of Nrf2, which suggests a role for this pathway in the EGCG-mediated induction of these endogenous antioxidant systems. Moreover, EGCG-mediated apoptosis was reduced by 50% with the inclusion of exogenous catalase in this process. This result suggested that EGCG induces apoptosis by ROS-dependent mechanisms [101]. It has been shown that green tea EGCG might be effective at improving the symptoms and pathological conditions associated with autoimmune inflammatory diseases in several animal models. The results showed that 50 mg/kg of body weight per day EGCG for four weeks increases myelin proteolipid protein (PLP) and oligodendrocyte transcription factor 1 (Olig1) expression in the cerebral cortex of cuprizone mouse model (C57BL/6 mice). PLP plays an important role in the formation or maintenance of the multilamellar structure of myelin, and Olig1 is a key regulatory factor of myelin specific gene expression and the program of myelinogenesis [102]. The combined application of EGCG and GA synergistically reduced cell death and promoted the axonal outgrowth of primary neurons. These effects could be translated into the EAE model, in which diminished clinical disease severity was associated with reduced CNS inflammation. There were no adverse interactions, and the two substances were used simultaneously; their use seems promising in clinical trials with MS patients [103]. A study carried out on female C57BL/6 mice of EGCG showed that dose-dependently EGCG ameliorated clinical symptoms and delayed disease onset in mice with EAE, which was well associated with reduced inflammatory infiltration and demyelination in the CNS. The mechanism underlying these effects was the altered regulation of CD4+T cell subsets (Th1/Th17 down-regulation, Treg up-regulation). Moreover, they also showed that EGCG reduced the production of IL-1β, IL-6, IL-17, IFN-β, and TNF-α. These results indicate that EGCG may attenuate the EAE autoimmune response by inhibiting immune cell infiltration and modulating the balance among pro-autoimmune and anti-autoimmune CD4+ T-cell subsets [104]. A randomized, double-blind, placebo-controlled crossover trial at the clinical research center on RRMS (*n* = 18) patients receiving glatiramer acetate (GA) showed that EGCG (600 mg per day) improves muscle metabolism during moderate exercise to a greater extent in men than in women, possibly because of sex-specific effects on autonomic and endocrine control [105].

### 4.7. Resveratrol

Resveratrol (trans-3,5,4’-trihydroxystilbene) is a naturally occurring polyphenolic compound that is found in more than 70 species of plants such as red grapes, cranberry, and peanuts. Resveratrol has been reported to demonstrate a range of activities including neuroprotection, anti-inflammatory, antioxidant, and anti-aging functions. The common recognition of resveratrol as a natural antioxidant is based on widely proven reports. There are studies that demonstrate that resveratrol may inhibit 5-LO and 15-LO in neutrophils, which produce varied pro-inflammatory metabolites during the arachidonic pathway [106]. Additionally, resveratrol is able to decrease the accumulation of hydroperoxides in LDL promoted by ferromyoglobin and the reduction of the oxoferryl complex to metmyoglobin, as well as prevent LDL modifications induced by peroxynitrite [107]. Some of the effects of resveratrol have been proposed to be exerted via the activation of sirtuin 1 (SIRT1), which is an NAD-dependent deacetylase, which prevents axonal degeneration [108]. Resveratrol has been found to induce the significant down-regulation of certain cytokines and chemokines in EAE-induced mice, such as TNF-α, IFN-γ, IL-2, IL-9, IL-12, and IL-17, as well as MIP-1α, MCP-1, and chemokine (C–C motif) ligand 5 (CCL5). Resveratrol has been shown to exhibit anti-inflammatory properties through a range of possible mechanisms, including: inhibiting the synthesis and release of pro-inflammatory mediators, modifying eicosanoid synthesis, or blocking iNOS and COX-2 pathways by inhibiting NFκB or AP-1. The up-regulation of Foxp3 gene expression is significant for the development and the differentiation of Tregs, and has also been observed in the splenocytes of mice treated with resveratrol compared with controls [109]. A pharmaceutical-grade formulation of resveratrol (SRT501) prevented neuronal loss during optic neuritis, which is an inflammatory optic nerve lesion in MS and EAE. SRT501 suppressed neurological dysfunction during EAE remission: it was found to induce a significant neuroprotective effect on SIRT1 activators in EAE mice, thus reducing neurological dysfunction during remission, and preventing axonal damage and neuronal loss. SIRT1 activators play a potential therapeutic role in preventing permanent neurological dysfunction in MS patients. Interestingly, neither SRT501 nor other SIRT1-activating compounds modulate inflammation in EAE, suggesting that resveratrol has the potential to complement current immunomodulatory MS therapies that fail to prevent neurodegeneration [110]. The potential role of resveratrol in the treatment of autoimmune diseases was evaluated using EAE-induced mice. It was shown that in an EAE animal model, resveratrol treatment decreased the clinical symptoms and inflammatory responses, which was mainly due to triggering apoptosis in activated T cells in the spinal cord and the reduction of pro-inflammatory mediators. These studies indicate the possibility of potentially using resveratrol in the treatment of inflammatory and autoimmune diseases, including MS [111].

### 4.8. Β-glucan

β-glucan is a naturally available polysaccharide that is found in the bran of cereal grains, where it is present in amounts of approximately 7% in barley, 5% in oats, 2% in rye, and less than 1% in wheat; it is also present in the cell wall of baker’s yeast as well as certain types of fungi [112]. β-1,3-glucan strengthens the immune system by enhancing the ability of macrophages, neutrophils, and natural killer cells to respond to and fight a wide range of invading organisms. Macrophages and dendritic cells are considered the major target of β-glucan, although neutrophils, B and T cells, and NKs are also known to be activated. β-glucan is a potent immunomodulator with effects on both innate and adaptive immunity. The cellular responses induced by β-glucan depend on their specific interactions with several cell surface receptors (PRRs) present on the immune cells, as scavenger receptors (SRs), lactosylceramide (LacCer), complement receptor 3 (CR3; CD11b/CD18), and dectin-1. Although the binding of β-glucan to LacCer or SRs on the cell surface of leukocytes has been described, the biological mechanisms that can result from these interactions and the effects on the immune responses are still lacking and need further investigation [113]. The binding of β-glucan to the dectin-1 receptor may induce the activation of many signaling pathways associated with innate immune responses, such as the production of inflammatory cytokines, the formation of ROS, and phagocytosis [114]. β-glucan can bind with Toll-like receptors (TLRs), especially TLR2/4, which is present on macrophages and stimulates them to produce cytokines, such as TNF-α and IL-12 via NFκB pathway signaling [115]. SRs are present on the epithelial cells, endothelial cells, and myeloid cells. SRs can recognize a variety of ligands including LDL and HDL (high-density lipoprotein). β-glucan can bind to SRs and activate multiple signals, such as phosphoinositide 3-kinase (PI3K), protein kinase B (PKB, also known as Akt kinase) and MAPK, which can activate plenty of signalization pathways [116]. Currently, β-glucan is studied for its anti-bacterial and anti-cancer properties; this molecule is considered to be a high immunomodulator. β-1,3-glucan consumption is recommended for all persons with a weakened immune system, those experiencing severe illness or recovering, or those who may be poorly nourished or exposed to the effects of electromagnetic fields or too large exposure to UVR [117]. Salim et al. reported prebiotic β-glucan supplementation to be associated with significant leukocytosis and lymphocytosis, hyperproteinemia, hyperglobulinemia, and significant decreases in triglyceride, total cholesterol, and glucose concentration, with no significant change in uric acid or creatinine concentration; in addition, significant increases were also observed in phagocytic activity and the phagocytic index [118]. Research conducted by Kogan et al. using the prepared water-soluble carboxymethylated (1,3)-β-d-glucan (CMG) showed that CMG exerted a potent protective effect against lipid peroxidation, which was comparable to that of the well-known antioxidants, such as D-mannitol and α-tocopherol. Additionally, its antioxidant activity was revealed at comparatively low concentrations (30 mM) [119]. There are many studies that have verified the potential role of β-glucan as a molecule that strongly affects the immune system [120,121]. However, it has never been studied in terms of its impact on the development of neurodegenerative diseases, including MS, in which the contribution of the immune system plays a crucial role. At the moment, there are only references from websites that indicate that supplementation with β-glucan helps in the MS treatment [122], but no epidemiological study has described an effect on MS course and potential molecular mechanisms.

## 5. Associations between Dietary Patterns and MS

It is well known that the intake of compounds such as vitamin D3, melatonin, or omega-3 PUFAs are increased in patients with MS. However, estimating the intake of bioactive compounds affecting the disease should be treated as a dietary pattern in general. The use of complementary and alternative medicine (CAM) have evidently risen in MS patients. CAM therapies include a wide variety of botanicals and nutritional products, such as dietary supplements (e.g., melatonin, omega-3 PUFAs), herbal supplements (e.g., curcumin, cannabinoids), and vitamins (e.g., vitamin A and vitamin D3) used instead of standard medical treatments or use simultaneously with the immunomodulatory drug therapy [123]. The literature reports the prevalence of CAM therapy use in MS varies from 33% to 80% [124,125,126]. The largest study was conducted in the United States (USA) by Nayak et al. on 3140 MS patients, which found that 64.9% implemented at least one CAM [127]. One category of CAMs is biologically based therapy, which includes using herb and nutritional supplements as part of general dietary patterns [128]. Studies in California and Massachusetts showed that 60% of MS patient respondents used CAM for treatment. Another study in British Columbia has shown that 67% of MS patients use CAM [129]. Research in 2003 on 20,778 MS patients enrolled in the North American Research Consortium on Multiple Sclerosis (NARCO MS) Patient Registry found that 54% of patients with MS used of at least one alternative medicine, of which 24% of them preferred to use additionally bioactive compounds in their nutrition [130]. The study conducted in 2009 on 428 MS patients in South Australia (SA) showed that the majority people with MS (64.7% of respondents) implemented CAMs/dietary interventions. The most frequently used CAM product categories were vitamins (81.8%), essential fatty acids (80.7%), and minerals (62.5%). Popular diets were low fat (39.8%), low/no sugar (23.8%), and gluten-free (16.4%) diets. The majority of those using CAMs/dietary interventions did so concurrently with conventional treatments (72.1%) [125]. A survey carried out in 2016 on 130 Eastern Turkey MS patients showed that 61.5% use of at least one form of CAM. The most common were herbs taken orally with rates of 55.5% [126].

The estimation of dietary patterns is a method to investigate the relationship between diet and disease, and determine the impact of food intake on the course of illness. Recent research has indicated that an increase in MS incidence may be due to environmental factors such as unhealthy dietary habits and poorly balanced diet. Diet patterns consist of a combination of plenty of nutritional components that are ingested simultaneously. The contribution of diet in disease development is of great interest to many with chronic diseases, including MS. The impact of the single component used in the diet cannot be unequivocally assessed. Therefore, is important to focus on the examination of the effects of universal used dietary patterns and rather than on the influence of the singular bioactive component [131]. Difficulties associated with chronic diseases, such as MS, have a negative impact on their quality of life and directly affect the limitation of their health-related activities. It is recognized that dietary interventions appear to have the potential profitable influence on the prognosis of MS. The analysis of dietary habits and the status of nutrition of patients with MS has never been widely studied; however, individual results suggest that many patients suffer from various forms of malnutrition [132]. Currently, there are 32 websites that provide advice or suggestions about using various dietary approaches to control the symptoms or progression of MS. The most recommended diets are a healthy balanced diet (low-fat, high-fiber diet with whole grains and fish), Swank diet (rich in low saturated fat), and modified Paleolithic diet (based on raw food, gluten-free, and lactose-free products), which primarily reduce fatigue and reduce the symptoms of MS [133]. The specific dietary patterns used by MS patients are generally characterized by diets that are low in saturated fat, processed meats, and refined sugars, and are rich in vegetables, fruits, nuts, and fish. In the available literature on the type of diet and supplementation, there are two main trends, which are derived from two researchers. Dr. Wahls based her theories on the hypothesis that the restriction of lectins would reduce intestinal permeability and CNS inflammation; furthermore, an increment of intake of specific food nutrients is key to neuronal health [134]. The Wahls diet was designed to reduce oxidative stress and excitotoxicity, nourish the mitochondria, prevent nutrient deficiencies, activate anti-inflammatory genes, and increase nerve growth factors [135]. The modified Paleolithic Wahls Elimination (Wahls Elim) diet generally emphasizes the consumption of leafy greens, vegetables rich in methionine and cysteine (e.g., cauliflower, Brussels sprouts, kale, black mustard seeds), colorful fruits, and nutrient compounds rich in PUFAs (e.g., extra virgin oil, walnuts, sunflower seeds, fish) [136]. There are several scientific reports suggesting that a modified version of the Paleolithic diet has been shown to reduce fatigue in both the SPMS and PPMS stages [137,138]. In 2009, Reese et al. carried out a case report of patient with SPMS that showed that a modified Paleolithic diet enhanced the recovery of this patient and led to an improvement in fatigue and a transition from wheelchair dependence do mild gilt disability [139]. Dr. Swank believed that MS had a vascular component and theorized that the restriction of saturated fat would reduce vascular dysfunction in the CNS. Swank’s diet was consistent with the cholesterol hypothesis of atherosclerosis, which implies saturated fat has a harmful effect on cerebrovascular health. The diet pattern was mainly based on low saturated fat (do not cross the limit ≤15 g saturated fat per day), fish, vegetables (e.g., avocado, olives), and supplementation of vitamin C and vitamin E. During the first year on the diet, red meat is not allowed, nor pork: only low-fat meats (e.g., lamb, rabbit, chicken liver) [140]. Swank’s studies have been criticized for not being randomized controlled trials; his comparison of responders may also be biased toward a positive result because patients who are feeling well are more likely to adhere to the saturated fat restriction. However, there are research studies that indicate positive associations between worsening disability and total cholesterol and triglycerides levels and negative associations between HDL cholesterol and the disability status of MS patients [141,142]. An increased intake of saturated fat is associated with increased recurrence in children (*n* = 219) with MS [143,144]. Although the Swank diet was developed 70 years ago, it is still in use. Prior to the development of the Wahls diet, in 2003, a survey of complementary and alternative medicine (CAM) use in 3140 individuals in the United States with MS found 16% of respondents following a Swank diet compared with 10.4% who were making other dietary changes [145]. Last year, a report was made on 6989 MS patients, in which 6.7% of people confirmed that they were using the Swank diet now or in the past [143]. The literature is very rich in reports confirming the impact of other diet patterns. Despite the use of classic diets, new studies are still being developed that attempt to verify the effect of other nutrients compounds on tMS. The results that are presented are sometimes very divergent. Some good examples of that phenomenon are included in the studies presented below. A cross-sectional study conducted by Moravejolahkami et al. was based on finding major dietary patterns and then evaluating the association between them and systematic inflammation, disease severity, intensity of fatigue, and anthropometric measurements. The Iranian (*n* = 26) participants with four subtypes of MS were divided into three groups. They discovered three dietary patterns for the study population: a diet rich in vegetables (e.g., cruciferous, onions, peppers curry, tomato, and squash), fruits (dried and fresh juice, and low-fat dairy products (FVL.patt), Mediterranean diet (MD) (MedL.patt), and Western-like dietary pattern (WestL.patt) described as high salt, red meat, sweets, and fast foods intake. The results that they obtained indicate that the MS patients groups that use the FVL.patt and MedL.patt had lower serum CRP levels, higher physical and mental health composite, and lower body mass index (BMI) scores and percent body fat compared to the WestL.patt group. Maintaining healthy eating patterns can significantly reduce systemic inflammation, reduce fatigue, and troublesome symptoms, improve the quality of life, and keep a balanced body mass in MS patients [146]. By contrast, research done 19 years earlier analyzed the diet among MS cases in the prospective Nurses’ Health Study (NHS) that consisted of 92,422 women (195 MS cases), interestingly, did not support these findings [147]. However, the latest research carried out eight years later on 185,000 women from the NHS (480 MS cases) in 2018 also confirmed no an association between the cumulative dietary scores and risk of MS. The effect of alternate Mediterranean diet was not significantly related to the risk of MS [148]. The retrospective case-control study was conducted by Dehghan and Ghaedi-Heidari among 120 patients with MS and 360 healthy individuals in Kerman, Iran. They showed that the diet used affects the risk of MS. The results revealed that vegetarian-only diets and animal-only diets were related to a higher risk of MS, in comparison with a mixed vegetarian–animal diet. Moreover, a vegetarian diet is rich in fiber, low calorie, and may reduce vitamin D input. Furthermore, this study showed the protective effects of consuming cow’s milk in infancy and the risky effect of vitamin D supplementation in developing MS, which is puzzling in view of the very large number of reports indicating its positive effect on MS [149,150,151]. The authors explained the results that the supplementation of vitamin D produces worse estimation criteria, while the measure of vitamin D in the serum gives more reliable results [152].

## 6. Conclusions

Randomized clinical trials seem to confirm the efficacy of some of the compounds discussed above, such as melatonin, vitamin D3, omega-3 PUFAs, and polyphenol compounds. However, further research is needed in order to understand the potential protective effects exerted by antioxidants on the cellular immunology of MS neurodegeneration. Such studies will not only result in an improved understanding of the disease’s mechanisms, but can also help with the implementation of proper dietary prophylaxis, and the establishment of new goals for innovative treatments providing real therapeutic benefits in MS. The interaction between medical treatment and active compounds contained in the diet should also be taken into account. The Food and Drug Administration (FDA) has approved several types of immunomodulatory pharmacological treatments for MS patients, including IFN-β, FTY, dimethyl fumarate (DMF), and GA. Currently, MS therapy cannot be firmly associated with a particular diet due to the scarcity of information on the effect of nutrition on the disease. Due to the numerous effects of the described compounds on the cells of the immune system and their influence on the production/activity of pro-inflammatory cytokines, possible interactions with the immunomodulatory drugs used in the treatment of MS should be considered.

## Figures and Tables

**Table 1:** Anti-oxidative properties of selected dietary compounds and standardized extracts used in the treatment of MS.

Compounds	Research Model	Dosage/Days/Criteria	Potential Role in MS	Ref.
**Curcumin**	**EAE** (*adult female Lewis rats*)	100 and 200 mg/kg/day per 14 days	- reduces clinical severity - decreases CNS inflammatory cells infiltration - inhibits neural Ag-specific T cell responses and IL-17 mRNA expression - suppresses gene expression of TGF-β, IL-6, IL-21 and STAT3 in spinal cord - inhibits phosphorylation of STAT3 in Jurkat T cells	[41]
**Curcumin**	**EAE** (*SJL/mice*)	50 and 100 μg /kg/day per 25 days	- inhibits of clinical and pathological symptoms of EAE - decreases CNS inflammation and demyelination in spinal cord - inhibits neural Ag-specific T cell responses - inhibits of MBP-specific T cell responses - inhibits IL-12 production in spleen cells, macrophages and microglia - inhibits IL-12-induced T cell responses - inhibits IL-12-induced tyrosine phosphorylation of STAT3, STAT4, JAK2 and TYK2 in T cells	[46]
**Polymerized form of nanocurcumin (PNC)**	**EAE** (*adult female Lewis rats*)	12.5 mg/kg/day per 29 days	- diminishes pro-inflammatory genes expression: MCP-1, IL-1, IL-17, NFκB in the lumbar spinal cord - augments expression of anti-inflammatory genes expression: IL-4, Foxp3, TGF-β in spinal cord - increases expression of oxidative stress marker genes: iNOS, HMOX-1 and Nrf2 in spinal cord	[47]
**Melatonin**	**Fetal rats**	10 mg/kg/day per 20 days	- increases gene expression of anti-oxidant enzymes: SOD and GPx in rat fetal brain	[57]
**Melatonin**	**SPMS** (*n = 16*)	10 mg per 30 days	- increases concentration of SOD and GPx in erythrocytes - decreases concentration of MDA in erythrocytes	[54]
**25(OH)D**	**MS**(*n = 256*)	MS patients >63.3 nmol/L	- diminishes the risk of MS (high circulating levels of vitamin D ae associated with a lower risk of MS)	[63]
**25(OH)D**	**MS** (*n = 196*)	MS patients ≥75 nmol/L	- decreases (approximately in 61%) risk of MS	[65]
**25(OH)D**	**MS** (*n = 196*)	MS patients ≥50 nmol/L	- enhances disease progression evaluated by MSSS and EDSS - lowers the NOx level in serum	[66]
**1,25-(OH)_2_D3**	**EAE** (*mice*)	0.5 μg/kg/day per 28 days	- down-regulates CYP24A1 gene expression in spinal cord (only in females)	[67]
**1,25-(OH)_2_D3**	**EAE** (*mice*)	50 ng/day (females)/100 ng/day (males)	- limits of occurrence of activated autoreactive T cells in the CNS	[68]
**1,25-(OH)_2_D3**	**EAE** (*mice*)	50 ng/day per 72 h	- reduces accumulation of macrophage in the CNS in spinal cord	[69]
**1,25-(OH)_2_D3**	**EAE** (*B10.PL(73NS)/Sn mice*)	200 ng (in 0.1 mL of soybean oil)	- stimulates inflammatory cell apoptosis, and enhances CNS cell survival in spinal cord	[70]
**1,25-(OH)_2_D3**	**EAE** (*mice*)	50 ng/day (females)/100 ng/day (males)	- acts directly on pathogenic CD4+ T cells and inhibits EAE via VDR in T lymphocytes	[71]
**1,25-(OH)_2_D3**	**RRMS** (*n = 50*)	0.5 μg/day for 12 months	- decreases relapses rate	[73]
**ESAPENT (*with 51% EPA and 31% DHA*)**	**MS** (*n = 20*)	6 g (in fish oil)/day for 6 months	- suppresses the capacity of monocytes to synthesize IL-1 and TNF-α - decreases in the population of the inflammatory cytokines: IL-1β, IL-2, TNF-α, and IFN-γ	[77]
**EPA and DHA**	**Healthy volunteers** (*PBMCs*)	10 μg/mL,25 μg/mL, and 50 μg/mL for 3 months	- decreases level of MMP-9 in PBMCs - inhibits MMP-9 activity - modulates immune cell production of MMP-9 - inhibits T cell migration	[78]
**EPA and DHA**	**MS** (*n = 80*)	EPA 16500 mg and DHA 4650 mg per 30 months	- no beneficial effect on MS patients	[80]
**TRIOMAR (*ω-3 fatty acids*)**	**MS** (*n = 102*)	EPA 270 mg and DHA 170 mg for 24 months	- no beneficial effect on MS patients	[81]
**RA**	**EAE** (*C57BL/6 mice*)	250 μg/kg/day	- inhibits the function of IL-17A-producing γ∆ T cells impairing their proliferation cytokine production and their pathogenic activity - inhibits cytokine production by Th17 cells - suppress IL-1R and IL-23R expression in γ∆ T cells	[85]
**Vitamin A**	**RRMS** (*n = 39*)	400 IU/day for 6 months	- increases expression of TGF-β PBMCs - increases expression of Foxp3 in PBMCs	[86]
**Flavonoid-rich extract (*FRE*)**	**Male Sprague–Dawley rats**	50-200 mg/kg/day per 7 days	- decreases expression of pro-inflammatory cytokines: NFκB, iNOS, COX-2, MMP-9, TNF-α in rats brain - diminishes level of p-ERK, MAPK, and phosphor-p38 in rats brain	[94]
**EGCG**	**EAE** (*Female SJL/J mice*)	300 μg/day per 131 days	- reduces EAE symptoms, brain inflammation, and neuronal damage in mouse brain - inhibits of TNF-α synthesis in T cells - decreases proliferation of CD4+ T cells	[98]
**Catechins**	**Healthy volunteers** (*n = 29*)	500 mg/kg/day for 4 weeks	- reduces plasma oxidized LDL by 18%	[99]
**EGCG**	**EAE** (*C57BL/6 mice*)	50 mg/kg/day for 4 weeks	- increases in PLP and Olig1 expression in cerebral cortex	[100]
**SRT501**	**EAE** (*SJL/J mice*)	100 mg/kg/day per 30 days	- attenuates neuronal damage and neurological dysfunction in EAE by a mechanism involving SIRT1 activation	[110]
**Resveratrol**	**EAE** (*C57BL/6 mice*)	100 mg/kg/day per 30 days	- decreases the clinical symptoms and inflammatory responses, mainly due to trigger apoptosis in activated T cells in spinal cord and reduces level of pro-inflammatory mediators.	[111]
**Β-glucan (*from baker’s yeast S. cerevisiae*)**	**Broiler chicks**	Total volume of carbohydrates from β-glucan 7.5 mg/mL daily per 21 days	- decreases in triglyceride, total cholesterol and glucose concentration (with no significant change in uric acid or creatinine concentration) - increases phagocytic activity and phagocytic index	[118]
**carboxymethylated (1,3)-β-d-glucan (CMG)**	**Male Lewis rat**	5 mg/kg/day per 28 days	- shows ability to protects against lipid peroxidation	[119]

Abbreviations: CMG – carboxymethylated (1,3)-β-d-glucan; CNS – central nervous system; COX-2 – cyclooxygenase 2; DHA – docosahexaenoic acid; EAE – experimental autoimmune encephalomyelitis; EDSS – expanded disability status scale; EGCG – epigallocatechin-3-gallate; EPA – eicosapentaenoic acid; Foxp3 – forkhead box P3; FRE – flavonoid-rich extract; GPx – glutathione peroxidase; HMOX-1 – heme oxygenase 1; IFN-γ – interferon-γ; iNOS – inducible nitric oxide synthase; JAK2 – Janus kinase 2; LDL – low density lipoprotein; MAPK – mitogen-activated protein kinase; MBP – myelin basic protein; MCP-1 – monocyte chemoattractant protein 1; MDA – malondialdehyde; MMP-9 – matrix metallopeptidase 9; MS – multiple sclerosis; MSS – multiple sclerosis severity score; NFκB – nuclear factor kappa B; Nrf2 – nuclear factor erythroid 2-related factor 2; Olig1 – oligodendrocyte transcription factor 1; PBMCs – peripheral blood mononuclear cells; p-ERK – protein kinase RNA-like endoplasmic reticulum kinase; PLP – proteolipid protein; PNC – polymerized form of nano-curcumin; RA – retinoic acid; SIRT1 – sirtuin 1; SOD – superoxide dismutase; STAT – signal transducer and activator of transcription; TGF-β – transforming growth factor β; TYK2 – tyrosine-protein kinase 2.
